# HighAltitudeOmicsDB, an integrated resource for high-altitude associated genes and proteins, networks and semantic-similarities

**DOI:** 10.1038/s41598-023-35792-3

**Published:** 2023-06-08

**Authors:** Apoorv Gupta, Sandhya Pathak, Rajeev Varshney, Yasmin Ahmad, Pankaj Khurana

**Affiliations:** Defence Institute of Physiology and Allied Sciences, Lucknow Road, Timarpur, New Delhi, 110054 India

**Keywords:** Data acquisition, Data integration, Data mining, Databases

## Abstract

Millions of people worldwide visit, live or work in the hypoxic environment encountered at high altitudes and it is important to understand the biomolecular responses to this stress. This would help design mitigation strategies for high altitude illnesses. In spite of a number of studies spanning over 100 years, still the complex mechanisms controlling acclimatization to hypoxia remain largely unknown. To identify potential diagnostic, therapeutic and predictive markers for HA stress, it is important to comprehensively compare and analyse these studies. Towards this goal, HighAltitudeOmicsDB is a unique resource that provides a comprehensive, curated, user-friendly and detailed compilation of various genes/proteins which have been experimentally validated to be associated with various HA conditions, their protein–protein interactions (PPIs) and gene ontology (GO) semantic similarities. For each database entry, HighAltitudeOmicsDB additionally stores the level of regulation (up/down-regulation), fold change, study control group, duration and altitude of exposure, tissue of expression, source organism, level of hypoxia, method of experimental validation, place/country of study, ethnicity, geographical location etc. The database also collates information on disease and drug association, tissue-specific expression level, GO and KEGG pathway associations. The web resource is a unique server platform that offers interactive PPI networks and GO semantic similarity matrices among the interactors.These unique features help to offer mechanistic insights into the disease pathology. Hence, HighAltitudeOmicsDBis a unique platform for researchers working in this area to explore, fetch, compare and analyse HA-associated genes/proteins, their PPI networks, and GO semantic similarities. The database is available at http://www.altitudeomicsdb.in.

## Introduction

A large percentage of the world’s population lives at High-Altitude (HA) areas and many also visit the mountains above 2500 m for outdoor activities such as trekking, climbing, and other adventure sports. Rapid ascent to high altitude leads to an instantaneous decrease in barometric pressure. The oxygen concentration remains the same but the number of oxygen molecules per breath is reduced; e.g. at an altitude of 3600 m, the barometric pressure decreases to 483 mmHg and < 40% of oxygen molecules are available to breathe.Since the amount of oxygen required for activity is the same, the body must adjust to having less oxygen or hypobaric hypoxia^1^. Some lowland residents adjust to the reduced oxygen availability at high altitude through a process known as acclimatization but some suffer from various disorders like Acute Mountain Sickness (AMS), High-Altitude Cerebral Edema (HACE), and High-Altitude Pulmonary Edema (HAPE) etc.^2,3^. Therefore research for the identification of the early signs of these physiological alterations is gaining momentum. A recent comparison in protein profiles of low-landers with their induction at high altitude has identified differentially expressed proteins like serum proteins Irisin, Myostatin, Acute Precursor Proteins (APPs), Apolipoprotein A1 etc. during HA acclimatization^4^. These proteins are associated with energy-related processes, skeletal muscle regeneration, inflammatory responses, and other hallmark molecular responses at high altitude^5,6^. Henceforth these proteins were proposed as biomarkers to predict early acclimatization of individuals at high altitude. Hunting for novel protein biomarkers in low landers and native samples using peptide profiling has become an important method to identify potential diagnostic or therapeutic markers^3,6^. Identification of the differentially expressed proteins that play a key role in the acclimatization process has helped to uncover the mechanisms responsible for the acclimatization at HA.A genome-wide study has uncovered plasma proteins that have the potential to predict vascular homeostasis during HAPE^7^. Similarly, a transcriptomic study indicated the modulation of multiple pathways and proteins involved in the early phase of hypobaric hypoxia exposure like VIM, CORO1A, CD37, STMN1 etc.^8^. Though there is enormous literature available that have reported ‘-omics’ profiles of humans and animals exposed to high altitude; the real challenge remains to integrate all these studies to produce a holistic understanding of continuously evolving mechanisms involved in functional adaptations of cells, tissues and organs, as well as the whole organism in the high-altitude hypoxic environment. Hence, we developed HighAltitudeOmicsDB where all this scattered data is collected, curated, analyzed, and visualized. The database currently contains ~ 1300 protein associations that have been manually curated from peer-reviewed publications which have been experimentally proven to be regulated by HA stress. The database stores the association of each protein with HA-stress in terms of the level of regulation (up/down-regulation), fold change, the study control group, duration and altitude of exposure, tissue of expression, source organism, level of hypoxia, method of experimental validation, place/country of study, ethnicity, geographical location etc. The database also provides whether the protein has been experimentally proven to be associated as a HA-biomarker and provides a link to the corresponding publication. The database is also cross linked to other databases like such as protein official symbol, protein aliases, chromosomal location, length, Uniprot ID, Enzyme Commission (EC) Number, Protein Family Information (Pfam) ID, Protein DataBank (PDB) ID, The Integrative Protein Signature Database (InterPro) ID, Single Nucleotide Polymorphism Database (dbSNP) Id. The database also presents protein’s functional information like GO annotation and Kyoto Encyclopedia of Genes and Genomes (KEGG) pathways association; their association with other diseases and drugs. The database also provides protein–protein network interactions of each protein with its top-50 interacting partners. The network can be visualised interactively on the webserver. Additionally, HighAltitudeOmicsDB calculates gene semantic similarity with these 50 interactors to identify functionally related proteins. The database additionally stores the transcription factors interacting with the gene and their regulation type (repression, activation, distal, proximal etc.). Additionally, the miRNAs interacting with the gene is also listed. Thus, HighAltitudeOmicsDB is a unique integrated platform to explore, retrieve, compare and evaluate genes/proteins associated with HA-stress, their PPI networks and semantic similarity and regulation by transcription factors and miRNAs. This will help uncover the underlying crosstalk between proteins that exists to acclimatize to HA and also provide mechanistic insights in these complex molecular responses. It will thus be useful in identifying novel and robust molecular biomarker candidates that can further help in the development of new diagnostic, prognostic and therapeutic strategies for high altitude disorders.

## Methodology

### Data collection

A combination of various keywords such as “high altitude”, “protein”, “gene”, “omics”, “hypobaric-hypoxia”, “anoxia” were used for extensive literature mining from PubMed and google search engines^9^. The publications were manually scrutinised to identify differentially expressed genes/proteins. After removing redundancy and duplicity, a comprehensive list of proteins that have been found to be Differentially Expressed (DE) at HA were curated from these publications. For each DE protein, its associated information was also fetched that includes; ‘Name of the protein’, ‘Protein Official Symbol’, ‘Aliases’, homologous ‘Human Entrez ID’, ‘Source Organism’, ‘Tissue of expression’, ‘level of hypoxia’, ‘altitude’, ‘duration of experiment’, ‘Level of regulation’, ‘Fold change’, ‘Experiment details’, ‘geographical location’, ‘ethnicity’, ‘Control group’, ‘Associated as Biomarker’. Studies in which the source organism was other than human, the homologous human gene/protein was identified using protein BLAST against the Uniprot Database. The homologous human protein with the highest sequence similarity and least E-score was selected. The minimum threshold was considered as > 80% pair wise sequence similarity. This way, even for experiments conducted on different experimental organisms (mice/rats/yak/bird/toad/sheep), human equivalence/translation would be easier.The collection was stored in JavaScript Object Notation (JSON) file format and stored in MongoDB^10^.

### Data processing and enrichment

For each protein additional details like protein official symbol, protein aliases, chromosomal location, length, Uniprot ID^11^, Enzyme Commission (EC) Number^12^, Protein family Information (Pfam) ID^13^, Protein Databank (PDB) ID^14^, The Integrative Protein Signature Database (InterPro) Id^15^, Single Nucleotide Polymorphism Database (dbSNP) Id^16^ was collected to help in cross-linking with other databases. For each protein, its GO-functional enrichment and pathway annotation were performed by Database for Annotation, Visualisation and Integrated Discovery (DAVID), and KEGG mapper tool respectively^17,18^.

For each protein in the database, its top-50 protein interactors were identified by the Search Tool for Retrieval of Interacting proteins (STRING) webserver^19^. The stringency for the search was kept at the highest level (0.9) and the filter was placed to allow a maximum of 50 associated proteins as the direct interactor of queried protein. STRING database constructs the protein–protein interactions network based on seven sources of information i.e. neighbourhood on the chromosome, gene fusion, phylogenetic co-occurrence, homology, co-expression, experimentally determined interaction, database annotated automated textmining. The interaction file was downloaded from the STRING database and was stored in JSON format.

To make the database more informative several other attributes were also added; protein-disease associations were mined from DisGeNET^20^; protein-drug relationship from DGIdb 3.0 database^21^. All these attributes were also stored in JSON files.

Gene Ontology (GO) annotation-based semantic comparisons between genes is an innovative approach to quantitatively assess the functional similarities between them. They have been extensively used across varied bioinformatics analyses^22,23^.The higher the semantic similarity score, more is the probability that two genes/proteins are likely to have a similar molecular function or be involved in a common biological process^22^. A low semantic similarity score shows two genes imparting different molecular functions. To identify semantic similarity, each protein in the HighAltitudeOmicsDB and its top-50 direct interacting proteins was submitted to the GOSemSim R algorithm^23^.GOSemSim is an R package for semantic similarity computation among GO terms, sets of GO terms, gene products, and gene clusters^23^. The results were represented in a 51 × 51 matrix. All these matrix files were also stored in the JSON file format.

### Database development

All constructed JSON files were transferred to the MongoDB database collection and uploaded to the server localhost using pymongo. Server query commands were made in the MongoDB compass. Vis.js library specifically was used to display the protein–protein interactions network^24^. The IDs such as Human Entrez ID, Uniprot ID, Protein Official Symbol, EC Number, PDB_ID, InterPro ID, Pfam ID, dbSNP ID, and reference PMIDs present in all tables are hyperlinked to the corresponding databases to provide additional details. The web interface also has a ‘Contact us’ page which includes a data submission form for the submission of any new data by the user. It would be reviewed and appended to the database on a regular basis.

## Results

### Web interface

HighAltitudeOmicsDB is a user-friendly, free-to-access resource that requires no prior registration. It is a comprehensive, non-redundant, manually curated resource of genes/proteins whose expression levels are experimentally validated to be associated with high-altitude stress. The database may be surveyed using “Browse” and “Search” options.

**Figure 1:**
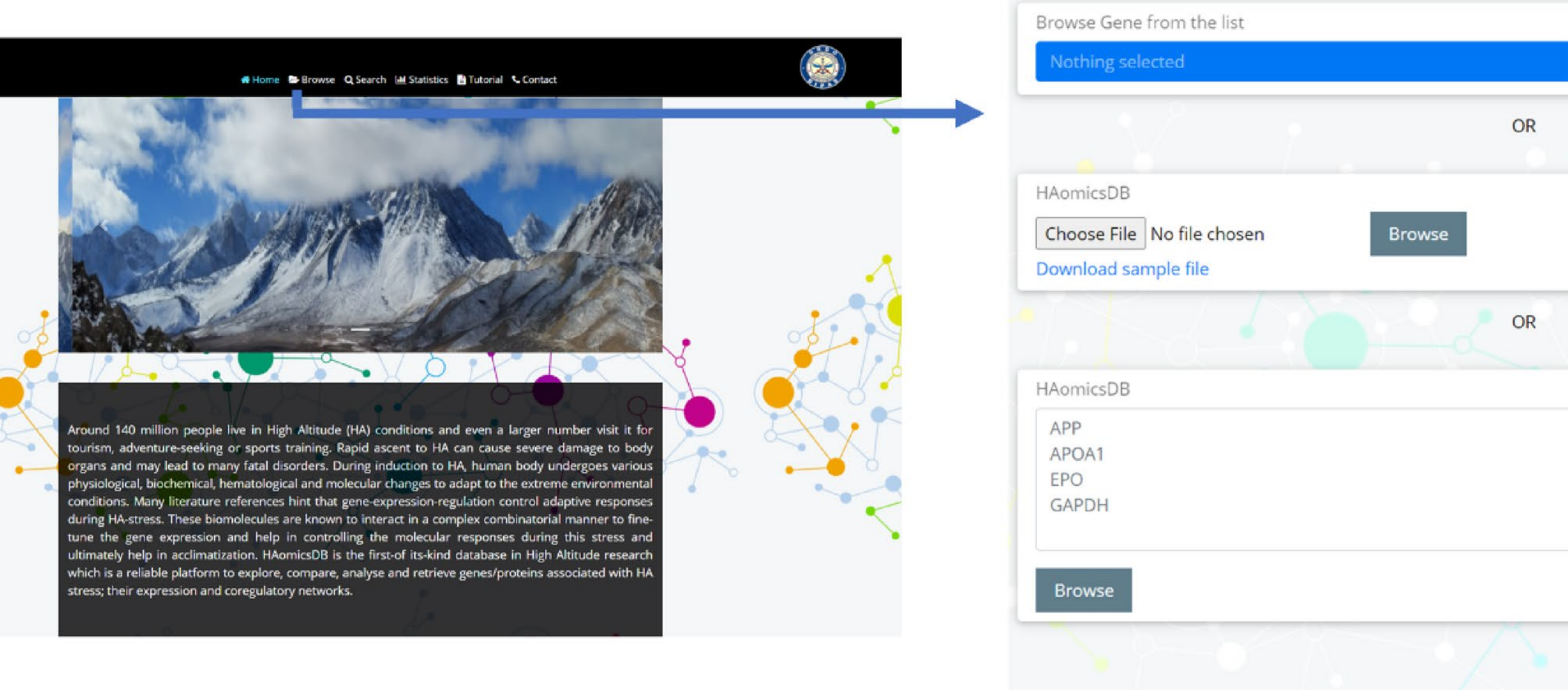
The home page and browse option of the webserver.

The “Browse” option allows the user to choose easily single or multiple genes/proteins from the database from a pull-down menu. Alternatively, the user may upload a file containing the protein official symbols or type the protein-official symbols. Clicking the adjacent ‘Browse’ button connects to a tabular format that hyperlinks the individual protein page. If the userlist contains protein symbols that are not in the database, a separate table highlighting the same is also provided (Fig. 1).

**Figure 2:**
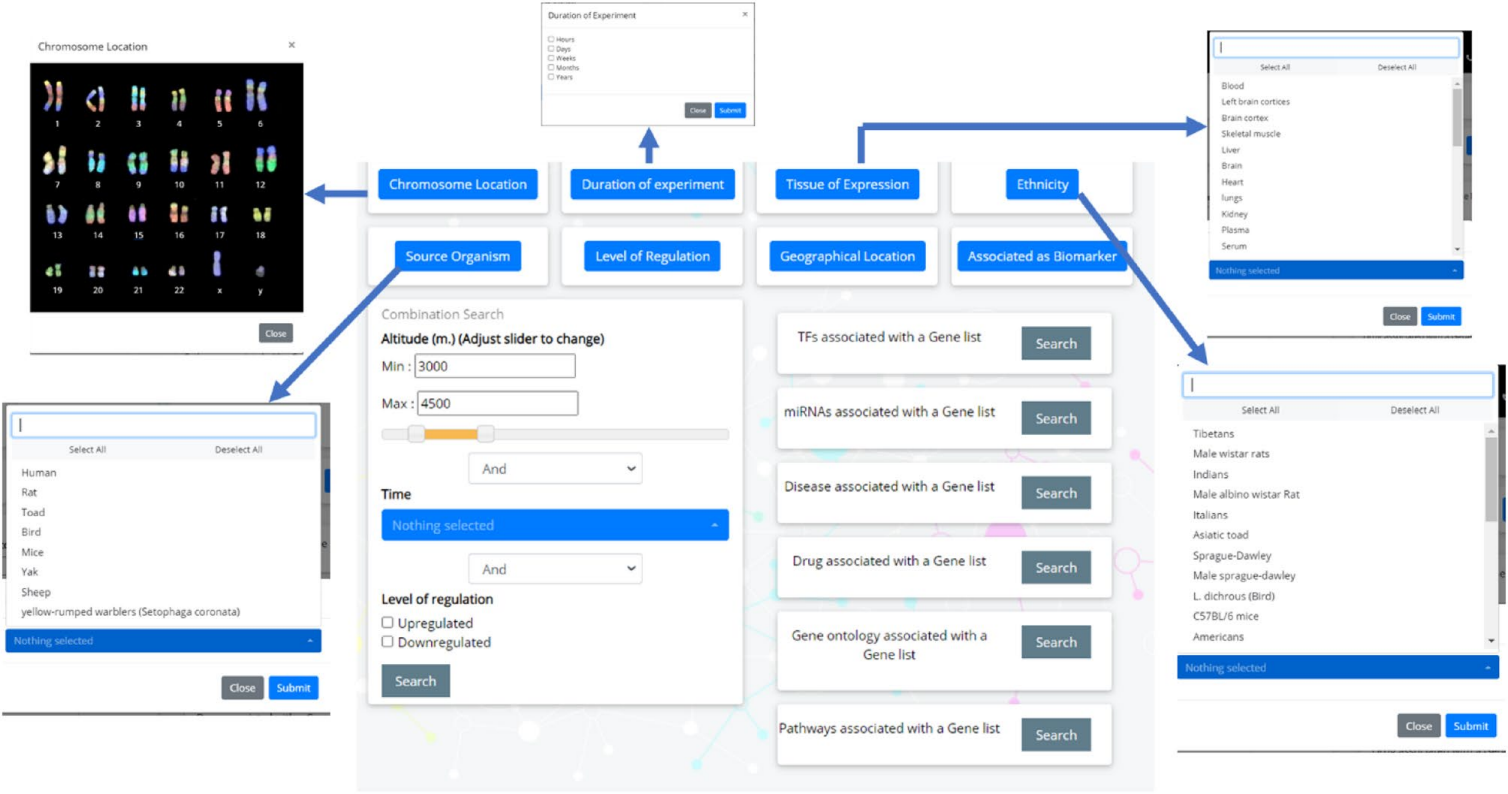
Screenshot of the “Search” module.

The “Search”option of the database offers multiple options to explore the database based on user research interests. Search by chromosome allows to click on any human chromosome number and identify the proteins of HighAltitudeOmicsDB which lie on the respective chromosome. Search by ‘duration of experiment’ allows identifying the list of genes/proteins whose expression changes in hours/days/weeks/months/years. Searching by ‘Tissue of expression’ opens a pull-down menu from which the user can choose the tissue of interest (Fig. 2). Searching by ‘Ethnicity’, ‘source organism’, ‘level of regulation’, ‘geographical location’ similarly opens a pull-down menu from which the user may choose the ethnicity, source organism, up/down-regulation, and location respectively, and get a tabular list of genes/proteins which are hyperlinked to the respective detailed information page of the protein (as discussed in following sections).

Additionally ‘Associated as Biomarker’ option leads to a tabular list of proteins that have been proposed/validated as molecular biomarkers for HA-stress. The protein symbols are hyperlinked to the respective protein page which provides a link to PubMed which validates the protein as a biomarker. Additionally to fetch proteins that are DE in an altitude-dependent manner, a user-interactive slider (ranging from 2200 to 9800 mt) is provided. The user may set the slider values and fetch genes/proteins which are associated with a defined altitude range. This has been combined with (AND/OR) options with the time of exposure to HA and level of regulation (Up/Down). The user may thus be able to make combination queries like up/down-regulated proteins expressed in days at an altitude range of 2200–4500 mt. The list of these proteins can be downloaded in Excel /PDF format for further analysis.

**Figure 3:**
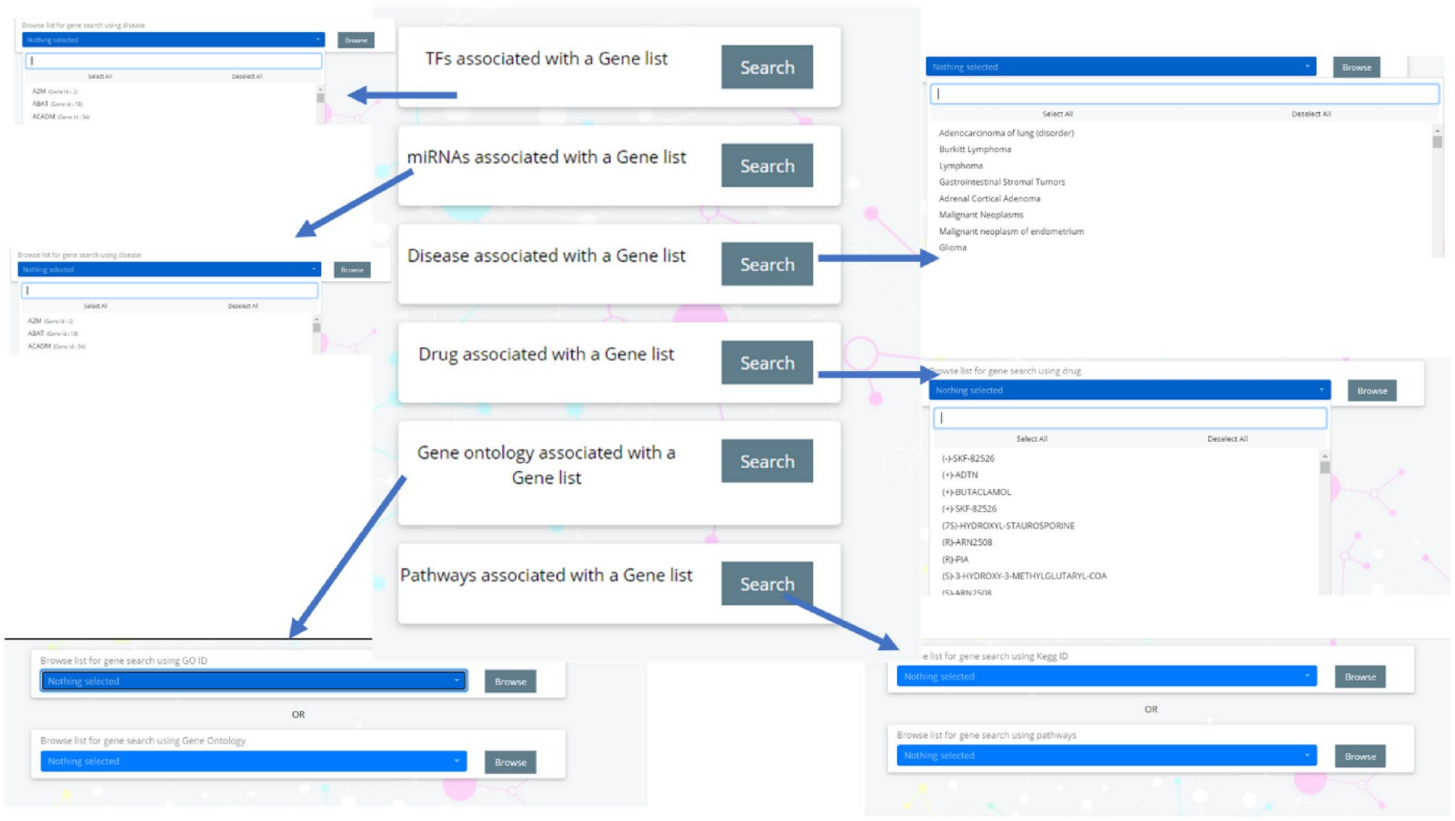
Screenshot of the “Search module”.

The webserver also allows to explore the proteins of HighAltitudeOmicsDB associated with a particular Transcription Factor (TF), miRNA, disease, drug, GO or KEGG pathway (Fig. 3).

**Figure 4:**
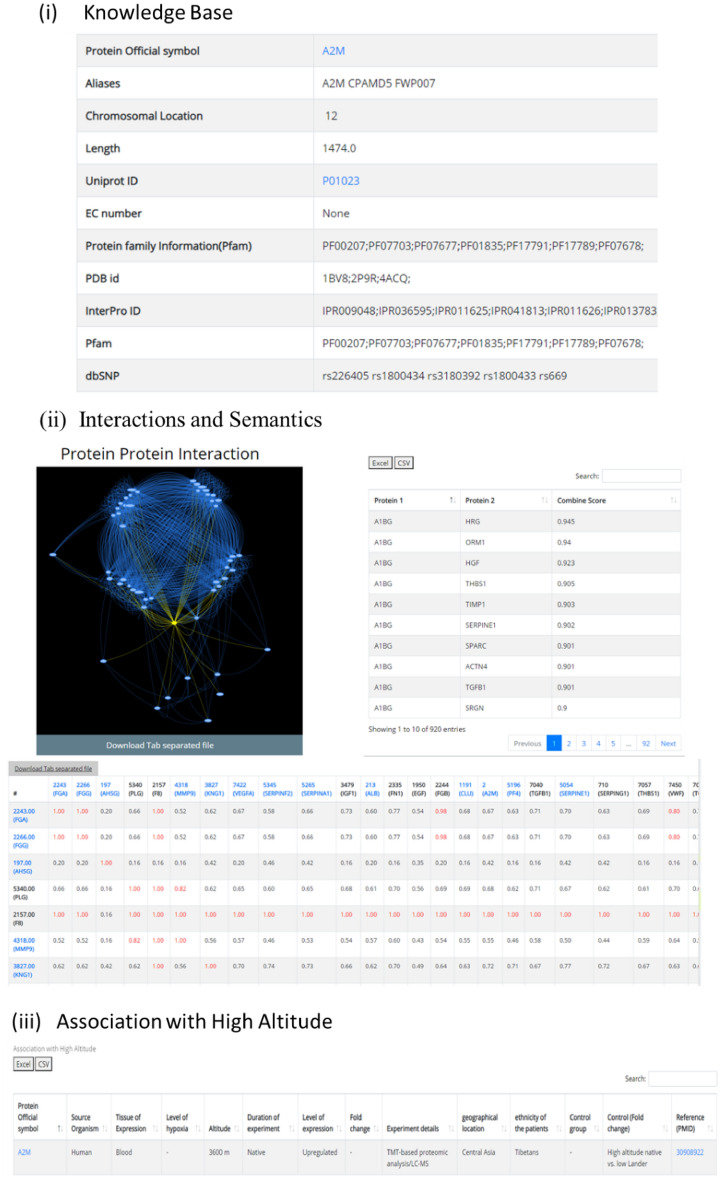
Protein information page details.

The details of the protein and its association with HA are provided in the detailed information page which may be divided into six sections (Fig. 4).

(i) Knowledge base

This is the first section of the database that gives general information about the protein like Protein Official Symbol, Aliases, Chromosomal location, Length, Uniprot ID, EC number, Pfam ID, PDB ID, InterPro ID, dbSNP ID which allows cross-linking to additional databases easily and quickly. The Uniprot ID is hyperlinked to the Uniprot Database.

(ii) Interactions and semantics

The top-50 direct protein interactors of each protein are identified from the STRING database using cut-offs described in the methodology section. The network is displayed in a user-interactive format with translation, zoom-in, and zoom-out features. The nodes are color-coded (yellow: the protein being studied; blue: the top-50 interactors). The edges are also color-coded (yellow: interactions between the protein being studied and its 50 direct interactors; blue: interactions among the top-50 interactors). The network may easily be downloaded in .sif format which can be easily visualised in network visualisation software like Cytoscape, Bina, etc. The list of interactions between them and their combined score is readily provided in a tabular format which can be downloaded in Excel/PDF format. The table is also provided with a ‘search’ option to easily search the protein of interest.

The pairwise GO semantic similarity score was calculated between the protein being studied and its top-50 interacting proteins as described in the methodology section. The results are visualised as a 51 × 51 matrix. The GO semanticsimilarity score > 0.8 is highlighted in red colour in the matrix. If any protein among the top-50 interactors is also a part of HighAltitudeOmicsDB, the protein symbol in the matrix is hyperlinked to the respective detailed protein information page within the database.This helps to identify any functional hubs of proteins that would be associated with HA stress and hence could shed light on the molecular basis for acclimatization/adaptation.

(iii) Association with high altitude

For each protein, its association with HA stress is compiled in a tabular format. The details are presented as the human protein symbol, source organism (organism in which the study was performed), tissue of expression, level of hypoxia, altitude, duration of the experiment, level of expression, fold change, experiment details, geographical location, ethnicity, control group expression, control group details and reference paper.The control group is defined based on the study plan, e.g. some studies had lowlanders as control groups and differentially expressed proteins were identified in HA-natives or lowlanders-who-ascent-to-HA. In some other studies, HA-natives were considered as controls and differentially expressed proteins were identified in lowlanders. So the control group varies as per the study and have been clearly mentioned in the database.The association of the protein as a biomarker is also compiled i.e., if the protein is ever been experimentally validated to be a biomarker, the entry in the column will be “Yes” otherwise “No”. A hyperlink to the respective publication which proves this association is also readily provided. The expression changes of a protein in different durations, tissues, and altitude conditions can be easily explored, compared, and analysed in this format.

(iv) Association with TFs and miRNAs

**Figure 5:**
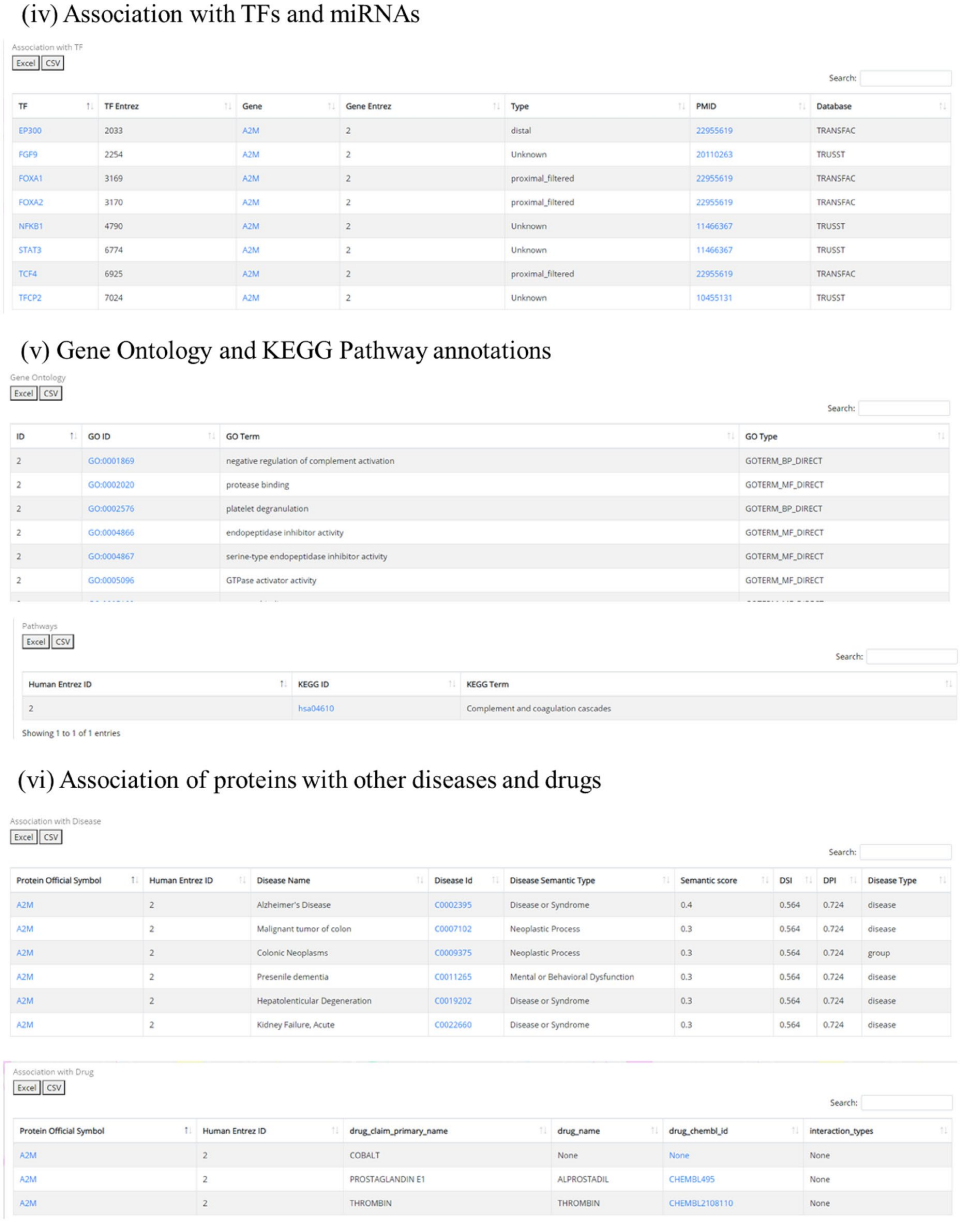
Screenshot of the protein information page.

Transcription Factors and miRNAs are the two most important transcriptional and post-transcriptional regulatory molecules fine-tuning the expression of genes. Thus the list of TF and miRNAs that are known the regulate the protein being studied is presented in a tabular format. The TF association table lists the TF symbol (hyperlinked to Genecards Database), its Entrez ID, symbol and Entrez id of the protein being studied, type of association, link to publication which ascertained this association and the database from which the association is extracted. The tables are downloadable in Excel/CSV format and provided with a ‘search’ option to explore the table with a user-defined keyword (Fig. 5).

Similarly, the miRNA-gene association table lists the miRNA miRTarBase ID, miRNA, symbol and Entrez ID of the protein being studied, experiment (luciferase reporter assay/western blot/PCR/Immunohistochemistry etc.), support type and link to respective publication (hyperlinked to PubMed) which ascertained this association. The tables may be downloaded in Excel/CSV format. The table is also provided with a ‘search’ option to explore the table with a user-defined keyword.

(v) Gene Ontology and KEGG pathway annotations

The Gene Ontology annotations are presented in a tabular format. The GO ID, GO Term, and GO Type are listed. The GO ID is also hyperlinked to QuickGO which provides detailed GO annotations^25^. The KEGG pathway annotations are also compiled and presented as KEGG ID and KEGG Term. The KEGG ID is hyperlinked to the KEGG database that provides additional details about the respective pathways.

Both these tables can be downloaded in Excel/CSV format and have an in-built ‘search’ option for keyword search.

(vi) Association of proteins with other diseases and drugs

This section provides details of drug and disease association. The information is represented in the form of tables belonging to each category respectively (Fig. 4). The first table shows information about the gene-target and its associated drug. This type of information can help the users to guide/design any gene/protein-based drug-targeting experiment. These two tables are equipped with the “search” option which helps in easy search of user-defined terms across lengthy tables. The tables can also be downloaded in Excel/PDF format.

### Web statistics

**Figure 6:**
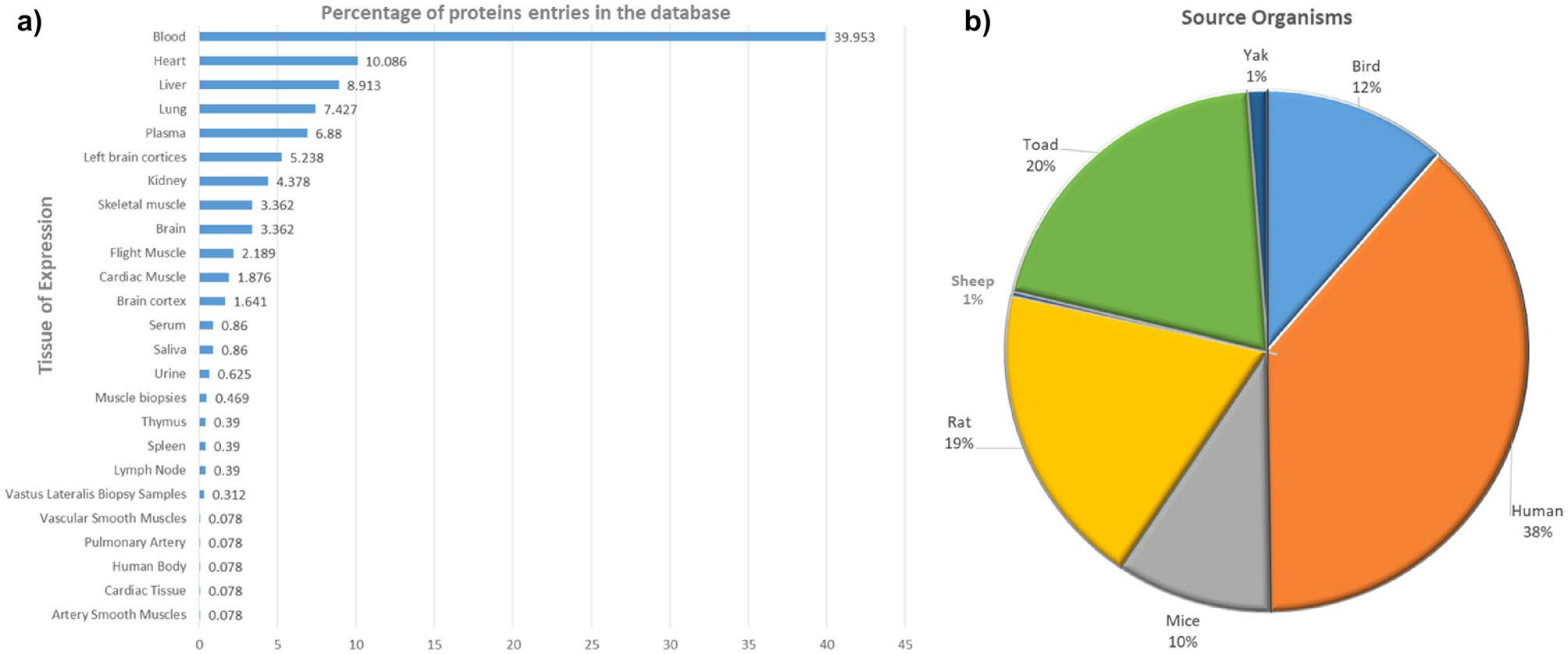
HighAltitudeOmicsDB Statistics (**a**) Distribution of high altitude proteins as per their tissue of expression. (**b**) Distribution of high altitude proteins studied in different source organism.

HighAltitudeOmicsDB contains ~ 1300 associations of 820 proteins that have been found differentially expressed at high altitudes. A detailed review of the database shows that all proteins were sourced from experimental studies in 25 tissues (Fig. 6a). These tissues are sourced from 7 animal species i.e. Human, Sheep, Rat, Mice, Yak, Bird, Toad (Fig. 6b). Humans as source organisms can be further characterised in terms of their ethnicity i.e. Americans, Tibetans, Han-Chinese, Italians, Nepali, Ladakhi, and Germans. The time of exposure is dependent on the source organism and it ranges from 0.5 h to 110 days for the native population.

**Figure 7:**
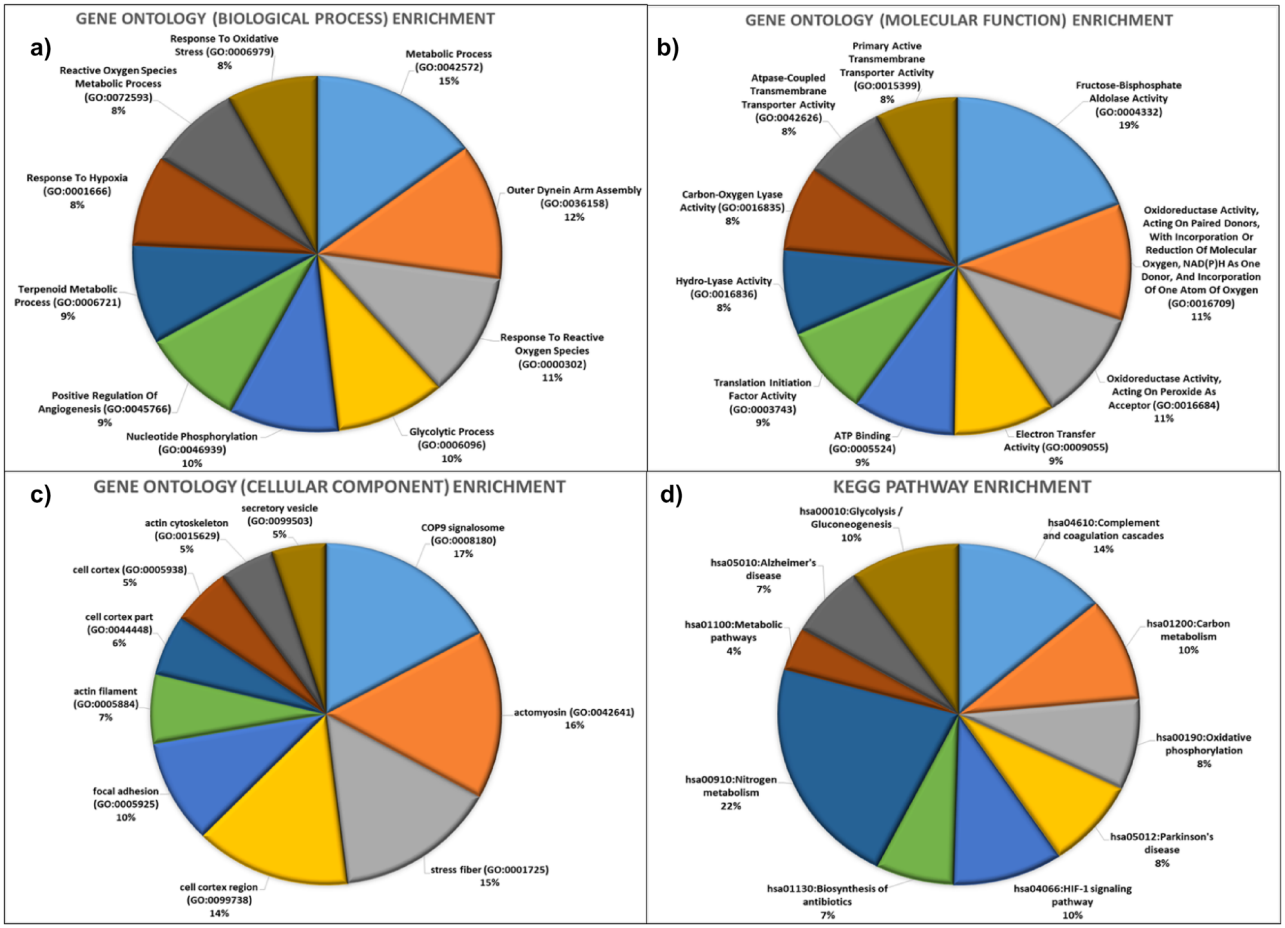
Functional characterization of differentially expressed High altitude proteins (**a**) GO: biological processes. (**b**) GO: molecular functions (**c**) GO: cellular compartment and (**d**) KEGG pathway enrichment.

The database contains two types of functional annotations- GO and KEGG pathway enrichment. The GO enrichment shows ‘Metabolic Process’ (GO: 0,042,572), ‘Outer Dynein Arm Assembly’ (GO: 0,036,158), ‘Response To Reactive Oxygen Species’ (GO: 0,000,302) as the top biological processes (Fig. 7a). ‘Metabolic process’ is highly associated with weight loss due to the adaptation mechanism at high altitude^26^. At high altitude, induction of hypobaric hypoxia activates HIF protein that further regulates genes responsible for mediating changes in cellular metabolism/energetics leading to weight loss due to increase in energy expenditure^27^. The second biological process ‘Outer Dynein Arm Assembly’ is the process for axonemal assemblies. The increase in the length and density of axoneme-like cilia due to hypoxia has been associated with cell death^28^. Lastly, ‘Response To Reactive Oxygen Species’ is the reflection of the redox status of the cell, and disturbances in redox status due to hypobaric hypoxia can lead to oxidative stress and DNA damage^3^. Similarly, terms like ‘Fructose-Bisphosphate Aldolase Activity’, ‘Oxidoreductase Activity’, ‘Acting On Paired Donors’, ‘Incorporation Or Reduction Of Molecular Oxygen’, ‘Oxidoreductase Activity’, ‘Acting On Peroxide As Acceptor’, ‘Electron Transfer Activity’ and ‘ATP Binding’, etc. are found to be top molecular functions of proteins present in the database (Fig. 7b). All the molecular functions are direct steps or feedback mechanisms associated with oxidative phosphorylation (aerobic respiration). Recent clinical studies have revealed that high-landers have a high percentage of mitochondria in their gastrocnemius muscle tissue, which aids in adaptation to a high energy expenditure environment^29^. ‘COP9 signalosome’ and ‘Actomyosin’ are the two cellular components terms that are found most enriched in differentially expressed protein sets present in the database (Fig. 7c). COP9 signalosome is part of the ubiquitin proteasomal degradation complex that controls the expression of pVHL, HIF-1α, and other oxygen responsive transcription factors regulated during hypobaric hypoxia^30^. Whereas actomyosin is a cytoskeleton of actin-myosin fiber complex present in different muscle tissues like skeletal muscle. The muscle fiber-type composition of both adult animals and humans is markedly altered during chronic exposure to high altitude.

The KEGG pathway enrichment shows ‘hsa00910: Nitrogen metabolism’ as the most enriched pathway in the differentially expressed HA protein set (Fig. 7d). Nitrogen metabolism is a process of nitrogen oxides production and these oxides such as nitrous, nitrite, nitrate have been found to play important role in high altitude adaptation response^31^. Overall functional annotation revealed the association of proteins present in the database with hypobaric hypoxic stress responses, which supports the comprehensiveness and specificity of the database.

## Discussion

During high altitude ascent, the body undergoes to extreme environmental stresses like hypoxia, hypoxemia, cold stress etc. that lead to many physiological changes in the body for its acclimatization to HA stress. Unbalanced physiological changes can lead to abnormalities or diseases such as High Altitude Pulmonary Edema (HAPE), High Altitude Cerebral Edema (HACE), Cardiovascular Disorders (CVD), hypothermia, muscle atrophy and different types of metabolic disorders that could be life-threatening. To identify diagnostic, prognostic, or therapeutic targets, the various biomolecules that are regulated in HA stress must be studied holistically. Towards this goal, HighAltitudeOmicsDB provides a comprehensive ready-reference resource of gene/ protein expression studies associated with high altitude conditions. It contains information of about ~ 1300 protein associations for metanalysis catering not only high altitude associated maladies but similar extreme conditions like cold stress. The search helps user to filter the data based on both unique as well as combination of features. The user can choose features that are directly associated with high altitude or indirectly. The combination search helps to extract specific dataset and help in reducing stochasticity of data. All these features enhance the chances of comprehensive systematic review and metanalysis. The web-resource is not only a HA specific protein repository; it can perform unique analysis that can help in comparing and analysing genomic/transcriptomic/proteomic data. The database provides PPI network interactions of each protein with its top-50 interacting partners. These PPI networks have been found useful in illuminating the functional mechanisms for abnormities in HA conditions^32–34^. HighAltitudeOmicsDB could accelerate the identification of biomarker discovery through gene semantic similarity matrix analysis. Literature reports prove that semantic similarity is an important tool for biomarker identification especially at high altitude^35^.

## Conclusions

HighAltitudeOmicsDB is an interactive resource and a server platform that captures and organises knowledge for genes/proteins associated with HA stress. It provides a comprehensive view of different HA-related studies; offers the annotations and visualisation of PPI networks and semantic similarities associated with gene/protein in the database. HighAltitudeOmicsDB is the first repository of comprehensive, manually curated resource of differentially expressed HA genes/proteins that were fetched using text mining and extensive literature survey. The information enables the user to browse biomolecules based on different query filters in the database, i.e., level of expression; duration of the experiment; altitude and source organism. HighAltitudeOmicsDB also encompasses protein-associated information such as TF and miRNA regulation, protein-disease association, protein-drug association. Hence the information base of HighAltitudeOmicsDB is very large and facilitates the use of this information for designing directed experiments for validation. HighAltitudeOmicsDB also identifies PPIs for each protein in the database and calculates GO semantic similarity between them.This unique feature helps to identify specific hubs of interacting proteins related to HA stress.The analysis of PPI networks and similarities would enable the user to infer mechanistic insights during HA stress. The webserver also offers functional correlation of proteins. The functional correlation includes both GO enrichment and KEGG pathway enrichment. The protein associated data can be downloaded from the database in excel/PDF format for further analysis.

## Data Availability

The data in the database (represented as tables) are easily downloadable in Excel/CSV format on the webserver. Any further datasets used and/or analysed during the current study will be availablefrom the corresponding author on reasonable request.

## References

[1] Simonson TS (2015). Altitude adaptation: A glimpse through various lenses. High Alt. Med. Biol..

[2] Basnyat B, Murdoch DR (2003). High-altitude illness. Lancet.

[3] Ahmad Y (2013). An insight into the changes in human plasma proteome on adaptation to hypobaric hypoxia. PLoS ONE.

[4] Wang C (2018). Exploration of acute phase proteins and inflammatory cytokines in early stage diagnosis of acute mountain sickness. High Alt. Med. Biol..

[5] Sliwicka E (2017). Serum irisin and myostatin levels after 2 weeks of high-altitude climbing. PLoS ONE.

[6] Ahmad Y (2011). Identification of haptoglobin and apolipoprotein A-I as biomarkers for high altitude pulmonary edema. Funct Integr. Genom..

[7] Sharma M, Singh SB, Sarkar S (2014). Genome wide expression analysis suggests perturbation of vascular homeostasis during high altitude pulmonary edema. PLoS ONE.

[8] Gaur P (2020). Temporal transcriptome analysis suggest modulation of multiple pathways and gene network involved in cell-cell interaction during early phase of high altitude exposure. PLoS ONE.

[9] Fiorini N, Lipman DJ, Lu Z (2017). Towards PubMed 2.0. Elife.

[10] Fejes AP, Jones MJ, Kobor MS (2014). DaVIE: Database for the visualization and integration of epigenetic data. Front Genet..

[11] Pundir S (2016). UniProt tools. Curr. Protoc. Bioinform..

[12] Bairoch A (2000). The ENZYME database in 2000. Nucleic Acids Res..

[13] Finn RD (2014). Pfam: The protein families database. Nucleic Acids Res.

[14] Berman HM (2000). The protein data bank. Nucleic Acids Res.

[15] Hunter S (2009). InterPro: The integrative protein signature database. Nucleic Acids Res.

[16] Smigielski EM (2000). dbSNP: A database of single nucleotide polymorphisms. Nucleic Acids Res.

[17] Dennis G (2003). DAVID: Database for annotation, visualization, and integrated discovery. Genome Biol.

[18] Kanehisa M (2017). KEGG: New perspectives on genomes, pathways, diseases and drugs. Nucleic Acids Res.

[19] von Mering C, Huynen M, Jaeggi D, Schmidt S, Bork P, Snel B (2003). STRING: A database of predicted functional associations between proteins. Nucleic Acids Res..

[20] Pinero J (2017). DisGeNET: A comprehensive platform integrating information on human disease-associated genes and variants. Nucleic Acids Res.

[21] Cotto KC (2018). DGIdb 3.0: A redesign and expansion of the drug-gene interaction database. Nucleic Acids Res.

[22] Pesquita C (2017). Semantic similarity in the gene ontology. Methods Mol. Biol..

[23] Yu G (2010). GOSemSim: An R package for measuring semantic similarity among GO terms and gene products. Bioinformatics.

[24] Rosenthal SB (2018). Interactive network visualization in Jupyter notebooks: visJS2jupyter. Bioinformatics.

[25] Binns D (2009). QuickGO: A web-based tool for gene ontology searching. Bioinformatics.

[26] Moore LG (2017). Measuring high-altitude adaptation. J. Appl. Physiol. (1985).

[27] Palmer BF, Clegg DJ (2014). Ascent to altitude as a weight loss method: The good and bad of hypoxia inducible factor activation. Obesity (Silver Spring).

[28] Brown JM (2003). Hypoxia regulates assembly of cilia in suppressors of Tetrahymena lacking an intraflagellar transport subunit gene. Mol. Biol. Cell.

[29] Scott GR, Guo KH, Dawson NJ (2018). The mitochondrial basis for adaptive variation in aerobic performance in high-altitude deer mice. Integr. Comp. Biol..

[30] Mikus P, Zundel W (2005). COPing with hypoxia. Semin. Cell Dev. Biol..

[31] Levett DZ (2011). The role of nitrogen oxides in human adaptation to hypoxia. Sci. Rep..

[32] Lopez-Cortes A (2020). Clinical, genomics and networking analyses of a high-altitude native American Ecuadorian patient with congenital insensitivity to pain with anhidrosis: A case report. BMC Med. Genom..

[33] Zhao Y (2019). Comparative proteomic analysis of Tibetan pig spermatozoa at high and low altitudes. BMC Genom..

[34] Xin J (2020). Chromatin accessibility landscape and regulatory network of high-altitude hypoxia adaptation. Nat. Commun..

[35] Ahmad Y (2015). The proteome of hypobaric induced hypoxic lung: Insights from temporal proteomic profiling for biomarker discovery. Sci. Rep..

